# AddTag, a two-step approach with supporting software package that facilitates CRISPR/Cas-mediated precision genome editing

**DOI:** 10.1093/g3journal/jkab216

**Published:** 2021-06-21

**Authors:** Thaddeus D Seher, Namkha Nguyen, Diana Ramos, Priyanka Bapat, Clarissa J Nobile, Suzanne S Sindi, Aaron D Hernday

**Affiliations:** Quantitative and Systems Biology Graduate Program, University of California, Merced, Merced, CA 95343, USA; Quantitative and Systems Biology Graduate Program, University of California, Merced, Merced, CA 95343, USA; Department of Molecular and Cell Biology, School of Natural Sciences, University of California, Merced, Merced, CA 95343, USA; Quantitative and Systems Biology Graduate Program, University of California, Merced, Merced, CA 95343, USA; Department of Molecular and Cell Biology, School of Natural Sciences, University of California, Merced, Merced, CA 95343, USA; Health Sciences Research Institute, University of California, Merced, Merced, CA 95343, USA; Health Sciences Research Institute, University of California, Merced, Merced, CA 95343, USA; Department of Applied Mathematics, School of Natural Sciences, University of California, Merced, Merced, CA 95343, USA; Department of Molecular and Cell Biology, School of Natural Sciences, University of California, Merced, Merced, CA 95343, USA; Health Sciences Research Institute, University of California, Merced, Merced, CA 95343, USA

**Keywords:** Cas9, RNA-guided nuclease, homology-directed repair, genetic complementation, *Candida albicans*

## Abstract

CRISPR/Cas-induced genome editing is a powerful tool for genetic engineering, however, targeting constraints limit which loci are editable with this method. Since the length of a DNA sequence impacts the likelihood it overlaps a unique target site, precision editing of small genomic features with CRISPR/Cas remains an obstacle. We introduce a two-step genome editing strategy that virtually eliminates CRISPR/Cas targeting constraints and facilitates precision genome editing of elements as short as a single base-pair at virtually any locus in any organism that supports CRISPR/Cas-induced genome editing. Our two-step approach first replaces the locus of interest with an “AddTag” sequence, which is subsequently replaced with any engineered sequence, and thus circumvents the need for direct overlap with a unique CRISPR/Cas target site. In this study, we demonstrate the feasibility of our approach by editing transcription factor binding sites within *Candida albicans* that could not be targeted directly using the traditional gene-editing approach. We also demonstrate the utility of the AddTag approach for combinatorial genome editing and gene complementation analysis, and we present a software package that automates the design of AddTag editing.

## Introduction

RNA-guided nucleases (RGNs) such as Cas9 have revolutionized genome editing by enabling the targeted introduction of double-stranded breaks (DSBs) within the genomes of living organisms. These DSBs create a powerful selection for DNA repair, which can be harnessed to promote integration of engineered exogenous “donor” DNA (dDNA) sequences in place of a cut target site, resulting in precision genome edits such as insertions, deletions, or substitutions in the genomes of organisms that support efficient homology directed repair (HDR). Because RGNs can easily be directed to introduce DSBs at unique user-defined target loci through the use of synthetic guide RNAs (gRNAs), this system represents a powerful customizable platform for genome editing (Jinek *etal.* 2012; Hsu *etal.* 2014; Adli 2018). Certain constraints, however, limit the flexibility of this technology, particularly when attempting to edit short genomic features.

**Figure 1:**
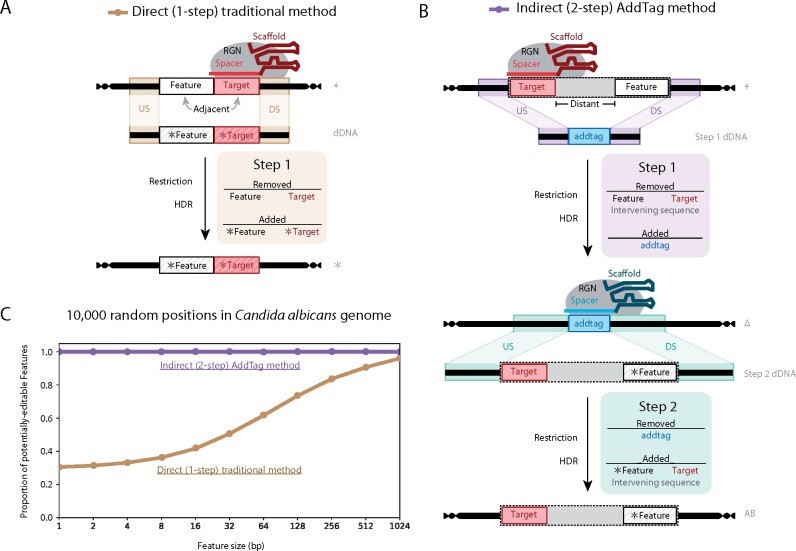
The two-step AddTag methodology enables precision genome editing of genetic loci that would not be possible using traditional (one-step) methods. (A) The direct (one-step) “traditional” method turns the reference (+) genome into the modified (*) genome by incorporating a single dDNA. Direct editing requires the RGN target site (target) to be disrupted. The modified target site (*target) must be sufficiently different from the target sequence to prevent RGN restriction with the same gRNA. Therefore, unless the genomic feature being edited (feature) and the target are largely overlapping, the final modified genomic sequence (*gDNA) must contain modifications outside of the modified feature (*feature). Any intervening sequence between the feature and target must also be short to ensure that the feature is replaced with the intended *feature. If the *target and *feature sites are not overlapping or closely adjacent, then HDR at the cut target site can result in *target incorporation without *feature incorporation (Supplementary Figure S10). (B) The indirect (two-step) AddTag method first turns the reference (+) genome into an intermediate (Δ) genome, and then turns the Δ genome into the add-back (AB) genome. AddTag enables precision feature editing without the need for a proximal target or any modifications outside of the intended *feature. Step one removes the feature and target, along with any intervening sequence, and replaces them with a unique engineered RGN target site (AddTag Target). Step two uses RGN cutting of the AddTag target to enable re-introduction of the previously removed intervening sequence (gray) and target, along with a modified *feature (or even the unmodified feature). Because the target sequence is not cut during step two, modifications to the target, or any other portion of the previously deleted locus, are not required. (C) Proportion of genomic loci that could potentially be edited via Direct (one-step) or Indirect (two-step, AddTag) methods, as a function of size. 10,000 *C. albicans* genomic loci were randomly selected, and the feature size at each locus was varied across 11 discrete sizes, ranging from 1 to 1024 bp (horizontal axis). The proportion of these 10,000 loci that could potentially be edited (vertical axis) via Direct (one-step) editing (tan line) versus Indirect (two-step, AddTag) editing (purple line) was assessed for each of the 11 discrete feature sizes. For Direct editing, sites were considered potentially editable if there was at least 1 bp of overlap between the feature and a Cas9 target motif ('N{17}|N{3}>NGG'). For Indirect editing, sites were considered potentially editable if a Cas9 target motif was found within a maximum expanded feature size of 4096 bp (See: Supplement—Identifying Targets and Feature expansion). For both Direct and Indirect editing, targets were required to pass the default AddTag quality controls: polyT ≤ 4.5, 25 ≤ GC ≤ 75, post-alignment Errors ≤ 5, Azimuth on-target ≥ 45, and Hsu-Zhang off-target ≥ 90. Thick horizontal lines represent DNA, with genomic DNA (gDNA) terminating in helices, and donor DNA (dDNA) terminating in blunt ends. Rectangles with internal labels represent annotated regions. Annotations with striped shading and labels preceded by an asterisk (*) represent modified sequences. The target sites that correspond to the reference (+) genome are colored red. The Spacer regions of the guide RNAs (spacer and scaffold) are color-matched with their complementary genomic DNA targets. Stretched rectangles labeled with upstream (US) or downstream (DS) indicate regions of homology between different DNA molecules and represent the intended recombination events during homology-directed repair (HDR). Vertical black arrows represent RGN-mediated cutting of the target locus, followed by dDNA incorporation via HDR.

The primary constraint that limits the flexibility of RGN-mediated genome editing is the need for a specific protospacer adjacent motif (PAM) sequence, which is recognized directly by the RGN protein, to be immediately adjacent to the user-defined target site (Jinek *etal.* 2012; Anders *etal.* 2014; Satomura *etal.* 2017). For example, the commonly used *Streptococcus pyogenes* Cas9 protein recognizes a 3’-adjacent NGG PAM sequence, thus limiting the extent to which A/T-rich sequences, such as noncoding DNA regions, can be targeted. Furthermore, the engineered dDNA must lack the user-defined RGN target site sequence (defined by the synthetic gRNA and adjacent PAM sequence) in order to prevent repeated cutting of the repaired target locus (DiCarlo *etal.* 2013), which would otherwise result in uncontrolled mutations via nonhomologous end joining rather than the intended precision editing via HDR (Supplementary Figures S1 and S2) (Mans *etal.* 2015). Essentially, RGN-mediated genome editing requires that the genomic feature to be modified contains or substantially overlaps the user-defined RGN target site, and the intended genome edit(s) must result in the ablation or substantial modification of the target site sequence (Figure 1A) (Horwitz *etal.* 2015; Mans *etal.* 2015; Biot-Pelletier and Martin 2016). While these constraints are generally not significant in the context of deleting or inserting large genetic elements, such as entire genes, they substantially limit the number of loci that can be modified with small-scale edits.

Two-step genome editing methodologies, which rely on the generation of intermediate genomic states en route to desired genetic modifications, have been developed to bypass RGN-mediated genome editing limitations in organisms ranging from *Escherichia* *coli* to mammals (Elison *etal.* 2017; Kwart *etal.* 2017; Zhang *etal.* 2017; Ikeda *etal.* 2018; Li *etal.* 2018; Feng *etal.* 2021). These approaches often require complex DNA cassette construction for each genetic modification of interest and/or the use of positive and negative selectable markers, and many of the “markerless” genome editing systems designed for use in prokaryotes are not feasible for use in diploid organisms. In addition, these approaches lack an integrated software package to facilitate implementation of the methodology. We present AddTag, a powerful two-step genome editing method with an integrated software package that bypasses the targeting constraints that limit traditional (one-step) RGN-mediated genome editing approaches (Figure 1B) and thus enables precision editing of virtually any genetic locus, independent of its size (Figure 1C). In two-step editing, the genomic feature to be edited does not need to overlap a unique RGN target site; instead, a user can utilize any potential RGN target site that is within the vicinity of the feature to be edited (Figure 1B). In the first step, an RGN is directed to cut at a user-defined target sequence that is near the genomic feature to be edited, and both the RGN target and the genomic feature, along with any intervening sequence, are replaced by a unique “AddTag” RGN target sequence. In the second step, RGN-mediated cutting of the AddTag sequence enables the introduction of virtually any DNA sequence of choice in place of the genomic region that was originally deleted in the first step. By decoupling the feature to be edited from the RGN target site, and thus removing the need to ablate the original RGN target site, this two-step methodology virtually eliminates traditional RGN targeting constraints and enables genome edits that would otherwise not be possible. This two-step methodology is effective at introducing small-scale edits into genomic features that cannot be directly targeted by RGNs. In addition, this general strategy also enables a wide range of reverse genetic approaches including the introduction of targeted deletion or substitution mutations, as well as the reintroduction of the gene of interest (*i.e.*, complementation).

**Figure 2:**
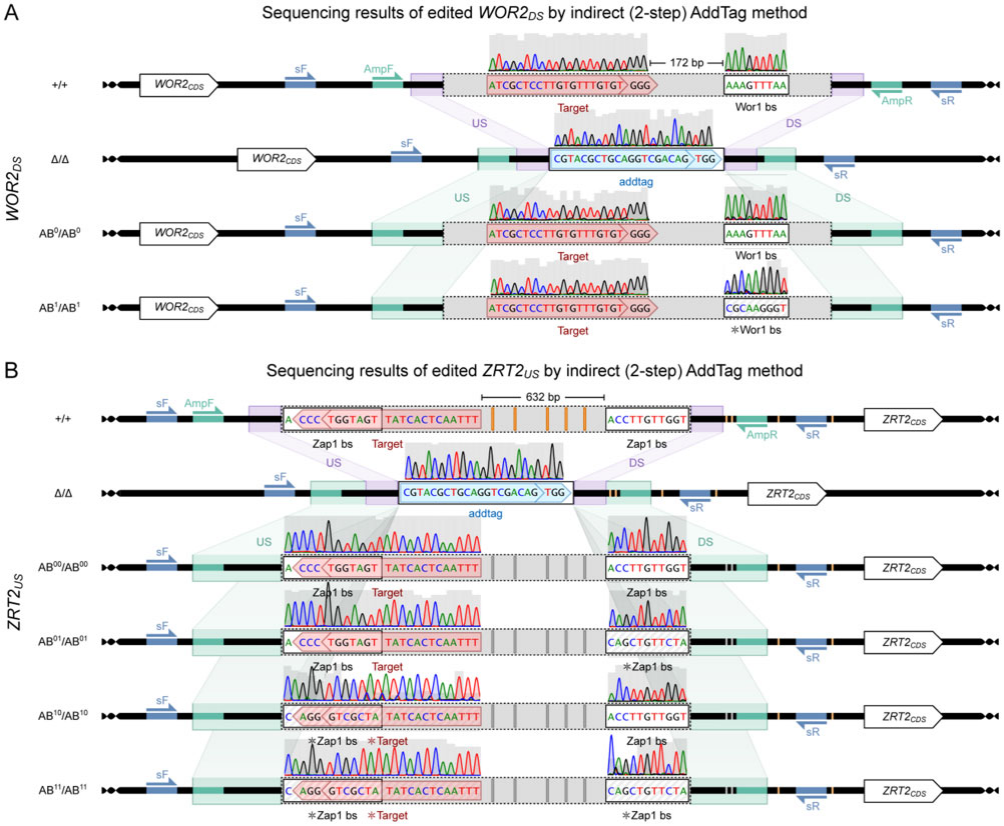
Precision editing of transcription factor binding sites enabled by the AddTag method. The AddTag method was used to edit short genomic features, including those that lacked overlapping RGN targets. Segments of Sanger sequencing chromatogram traces are depicted for the experimental target and feature at the edited locus, however, the entire region encompassing the step-2 addback dDNA, including the integrative flanks, was verified by Sanger sequencing for each modified strain depicted. Gray bars in the traces represent the Phred quality score from 0 (low) to 62 (high). For step one (purple), the wild-type (+/+) genome was turned into the intermediary (Δ/Δ). For step two (green), the intermediary genome is turned into an add-back (AB/AB) genome. (A) A 9 bp Wor1 binding site (Wor1 bs) that is located downstream of the *WOR2* coding sequence and lacks an overlapping RGN target site was edited via the AddTag method. In step one, both the Wor1 bs and an RGN target 172 bp upstream, along with intervening and flanking sequences included in the expanded feature, were replaced with an AddTag target to create the intermediate *wor2_DS_Δ/Δ* genotype. Two parallel step two transformations converted the intermediary genome into either an add-back genome (AB^0^/AB^0^) containing the wild-type Wor1 bs, or an add-back genome (AB^1^/AB^1^) containing an edited Wor1 bs. All sequences outside of the Wor1 bs that were deleted in step one were subsequently restored to their wild-type state in step two. (B) AddTag method was used to perform combinatorial editing of two 11 bp Zap1 binding sites (Zap1 bs) that are located 645 bp apart upstream of *ZRT2_CDS_*. In step one, the two Zap1 bs sequences, along with the intervening sequence, were replaced with an AddTag. Four parallel step two transformations produced add-back genomes with neither (AB^00^/AB^00^), either (AB^01^/AB^01^ and AB^10^/AB^10^), or both (AB^11^/AB^11^) Zap1 bs sequences edited. Genomic positions within the feature and homology arms containing heterozygous allelic variants (orange) in the wild-type genomic DNA (+/+) became fixed in the homozygous state (dark gray) in each add-back genome (AB). Thick horizontal lines represent DNA, with genomic DNA (gDNA) terminating in helices. Rectangles with internal labels represent annotated regions. Annotations with striped shading and labels preceded by an asterisk (□) represent modified sequences. Homologous regions on different DNA molecules are connected by stretched rectangles and represent intended recombination events. The sequence intervening between the feature selected for editing and the restriction target is colored gray. Half-arrows pointing right represent annealing of “forward” primers, and half-arrows pointing left denote annealing of “reverse” primers. Genomic sites where primers anneal are color-matched to their respective primers.

**Figure 3:**
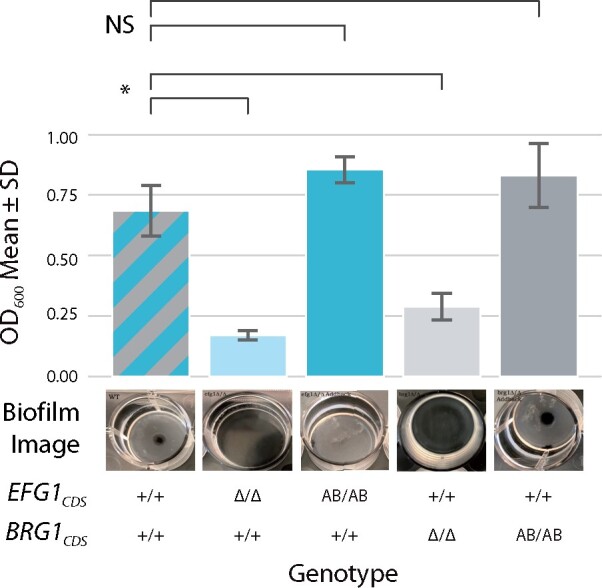
AddTag-mediated homozygous gene restoration at native loci confers full complementation of the wild-type phenotype. Strains with *EFG1* or *BRG1* restored at their native loci are indistinguishable from the original wildtype strain background in which the *efg1* or *brg1* deletion strains were engineered. Each column represents a different genotype, with a representative image and the OD_600_ of its biofilm depicted as a bar above. For each genotype, two independently derived strains were cultured in a 24-hour biofilm assay at *n* = 4 wells. NS: *P*-value > 0.05; *P-value < 0.05. For both *EFG1* and *BRG1* loci, the Δ/Δ genotype shows a biofilm growth defect, and the AB/AB genotype shows full phenotypic restoration.

**Figure 4:**
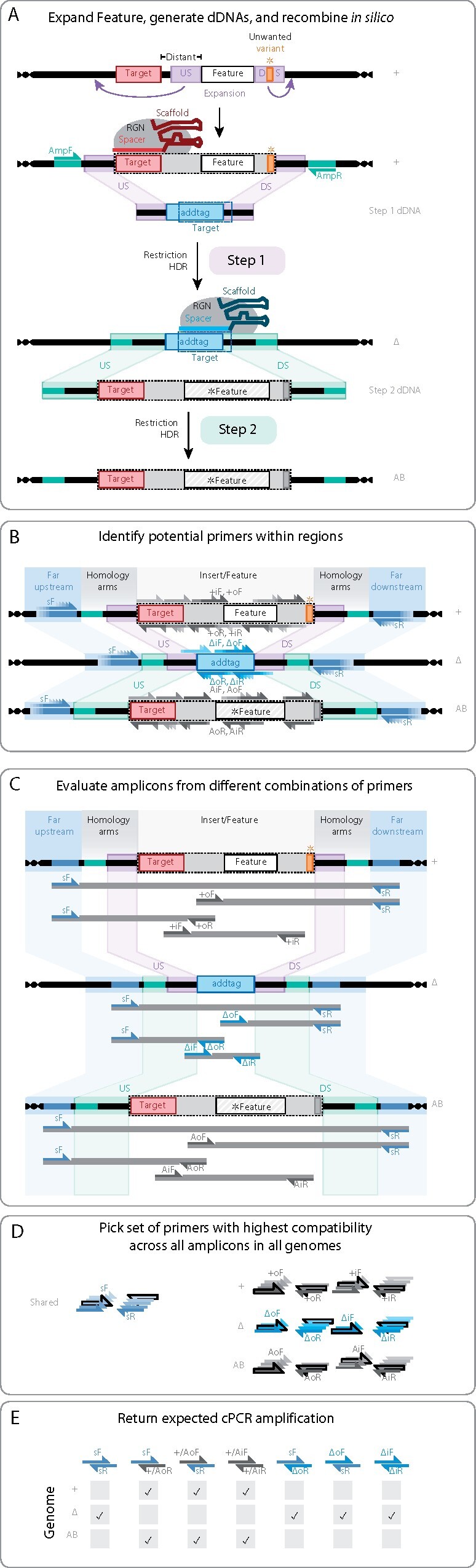
Overview of how AddTag software identifies targets, generates dDNAs, and chooses primer pairs for strain validation. AddTag software input requires a reference genome sequence (+) with the annotated feature to be edited, a *feature sequence with which to replace the wild-type feature, and one or more RGN target motifs, representing the specific RGN(s) being used for HDR-mediated genome editing. Each panel of this figure describes the internal process the AddTag software uses to produce its output. (A) The AddTag software automatically identifies and designs the gRNA target (red) and AddTag target (cyan) sequences used for RGN-mediated cutting of genomic DNA in steps one and two, respectively. If necessary, the AddTag software will expand the bounds of a user-defined feature to include an RGN target that meets or exceeds gRNA quality control filters. The feature expansion process also ensures that flanking homology arms manifest an acceptable level of polymorphism. In this example, the feature is expanded in both directions (violet arrows). The software also automatically generates the dDNA sequences used in steps one and two, based on specific user inputs and the outcome of the feature expansion process. Step one dDNA consists of upstream and downstream homology sequences (violet US and DS regions) derived from the reference gDNA (+gDNA) sequences that flank the expanded feature, combined with the AddTag insert to create a new RGN target (box with dark blue, dashed lines). Step two dDNA consists of expanded upstream and downstream homology regions (green US and DS regions) flanking the expanded genomic feature that was removed in step one (gray box with dashed line border). A wild-type version of the step two add-back dDNA (not shown) can be amplified from +gDNA using the output AmpF/AmpR primer pair (green), while step two dDNAs with modified sequences (*Feature) can be generated by stitching PCR or DNA synthesis (not shown). Vertical black arrows represent gRNA and RGN complex association and restriction of gDNA, followed by homology-directed repair (HDR) as the process by which dDNA is incorporated into the gDNA. (B) The AddTag software systematically searches for candidate primer sequences to populate a standardized set of PCR primers that can be used for dDNA amplification and genotype verification for each genome editing experiment. Some regions, such as the far upstream and far downstream (blue), are shared among all gDNAs (+, Δ, and AB genotypes). Other regions are genome-specific, like the AddTag target (cyan) and expanded feature (gray) regions. The software uses a sliding window approach to identify all potential primers within each region, but for simplicity only a few are depicted for each region. (C) The AddTag software identifies candidate primers that would be suitable for the generation of PCR amplicons indicated by dark gray bars. These include: the sF/sR primer pair, which spans all genomes (+, Δ, and AB gDNAs), and 3 primer pairs which are specific to each genome, labeled sF/oR, oF/sR, and iF/iR. The software assigns a weight to each primer, then it evaluates the compatibility of every pairwise combination of forward and reverse primers for each amplicon and assigns a weight to that primer pair (Supplement—Finding, scoring, and ranking primer designs). Note: if the (possibly expanded) feature or AddTag target are small, then a usable iF/iR primer pair might not be found for that gDNA. (D) The AddTag software selects an optimal integrated set of PCR primers from the pool of primer pairs identified in panel (C). Each color-coded stack of primers represents an arbitrarily large set of primers identified through the sliding window approach for that like-colored region in panel (B). Simulated annealing identifies the set of primer pairs with highest compatibility (black outline) (Supplement—Constructing optimal primer sets). (E) The AddTag software calculates expected cPCR amplification for each gDNA using *in silico* PCR. Given restrictive cPCR conditions (pictured) sF/sR pair is expected to amplify only the ΔgDNA, and fail to amplify the +gDNA and ABgDNA. Alternatively (unpictured), if the feature size is small, amplification should occur at all gDNAs and band migration on a gel should indicate the successful step one dDNA integration. Amplification of the sF/oR and oF/sR pairs across the gDNAs indicate the feature or insert is present at the expected locus (it is possible dDNA may incorporate at an unintended locus). In this example where none of the optimal primers overlap with the feature or *feature, several primers are identical: +/AoF (+oF and AoF), +/AoR (+oR and AoR), +/AiF(+iF and AiF), and +/AiR (+iR and AiR). The iF/iR pairs amplify if the feature or insert exists anywhere in the gDNA, regardless of its locus. Thick horizontal lines represent DNA, with genomic DNA (gDNA) terminating in helices, and donor DNA (dDNA) terminating in blunt ends. Rectangles with internal labels represent annotated regions. The target site that matches the wild-type is colored red. The Spacer regions of the guide RNAs (Spacer and Scaffold) are color-matched with genomic regions the RNA-guided nuclease (RGN) is programmed to cleave. Allelic variants on DNA are colored orange, and fixed variants are colored dark gray. Homologous regions on different DNA molecules are connected by stretched rectangles, and represent intended recombination events. Half-arrows pointing right represent annealing positions of “forward” primers, and half-arrows pointing left denote annealing positions of “reverse” primers.

To demonstrate the utility of the AddTag approach and integrated software package, we performed a series of genome edits in the diploid human fungal pathogen *Candida albicans*. We note that although we use *C. albicans* as our test case model organism due to prior expertise and availability of our previously published RGN-mediated genome editing system (Nguyen *etal.* 2017), our approach and software package should be amenable to other organisms with efficient RGN-mediated genome editing capabilities. First, we show that it is possible to edit small genomic features, such as transcription factor binding sites, that could not be edited using the traditional (one-step) approach due to RGN targeting constraints (Figure 2A). Second, we demonstrate that the AddTag approach can be used to easily generate a matrix of isogenic strains to investigate the effects of combinatorial mutations in neighboring genomic features (Figure 2B). We also highlight the advantage of using the AddTag approach for gene complementation analyses by completely restoring the wild-type phenotype of gene deletion strains where previous approaches had failed to achieve full phenotypic restoration (Figure 3). The custom software package that we provide automates the extensive manual design work that would otherwise be necessary to implement the AddTag approach to genome editing (Figure 4). Not only does this software automate RGN target selection and dDNA design for both steps of the AddTag approach, it also designs an integrated set of PCR primers for validation of the intended genome edits after each step (Figure 4E, Supplementary Figure S3). These features make the AddTag software unique in comparison to other utilities that are designed to support RGN-mediated genome editing (Supplementary Table S1).

## Materials and methods

### Plasmids and synthetic DNA

For all genetic modifications in this paper, we used the AddTag software to automatically select the RGN targets, dDNAs and corresponding AddTag targets for step one editing, determine optimal primers (AmpF/AmpR) for amplifying the step two dDNAs, and to pick cPCR primers for validating integration of the intended modified features at the target loci following each step of editing. The gRNA expression cassettes used to make all deletion and complementation/add-back strains were generated via an “all-in-one” PCR stitching approach (Supplementary Figure S4) (Supplement—“All-in-one” gRNA cassette stitching). Briefly, linear DNA fragments containing the pSNR52 promoter and the invariable structural component of the gRNA coding sequence were PCR amplified from pADH110 and pADH119, respectively, using AHO1096/AHO1098 and AHO1097/AHO1099 primer pairs and Phusion polymerase (ThermoFisher). The resulting fragments were stitched together in a single reaction using custom target sequence-specific bridging oligos and AHO1237/AHO1238 amplification primers. Linear Cas9 expression cassettes were generated by MssI digestion of pADH137, and were transformed along with the stitched custom gRNA expression cassettes and custom dDNA fragments. The step one dDNA fragments for the *ADE2*, *EFG1*, and *BRG1* loci were generated by annealing complementary 100-mer oligonucleotides. We used overlapping primer extension with Phusion polymerase to generate step one dDNAs for the *WOR1* and *ZRT2* loci. Wild-type add-back dDNA fragments were generated by standard PCR amplification of *C. albicans* genomic DNA using AddTag-designed amplification primers (AmpF/AmpR) and Phusion polymerase. Donor DNA fragments containing mutated Zap1 binding sites were first synthesized as full-length synthetic DNA fragments (ThermoFisher) then PCR amplified using the same AddTag-designed primers used to amplify the corresponding wild-type dDNA fragments. All DNA plasmids and primers used in this study are included in Table S2 and Table S3, respectively, and the base plasmids used in this study (pADH110, pADH119, and pADH137) and their annotated sequence files are available through Addgene.

### Cell culture and transformation

All *C. albicans* strains used in this study (Supplementary Table S4) were derived from strain SC5314. AHY940 (SC5314 with one allele of *LEU2* deleted) was used as the base strain for all genome editing procedures, and transformations were performed as previously described (Nguyen *etal.* 2017). Briefly, gRNA and Cas9 expression cassettes, along with dDNA fragments, were transformed into AHY940 or derivative strains via chemical transformation and plated onto YPD agar plates (2% Bacto peptone, 2% dextrose, 1% yeast extract, and 2.5% agar) supplemented with 200 µg/ml nourseothricin (NAT; GoldBio). Transformation plates were incubated for 2 days at 30°C to select for integration of the gRNA and Cas9 expression cassettes (Nguyen *etal.* 2017), and genome editing at the target locus was validated by colony PCR using AddTag-generated primers. Subsequent to genotype verification, the gRNA and Cas9 expression cassettes, along with the NAT resistance marker, were removed via the LEUpOUT method by selection on synthetic defined (SD) agar medium without leucine (Nguyen *etal.* 2017). Strains that harbored mutated Wor1 or Zap1 binding sites and their wild-type add-back counterparts were further validated at the base pair level via Sanger sequencing of colony PCR products that spanned the engineered loci (Figure 2, Supplementary Figure S5).

### Phenotypic assessment of Zap1 binding site mutant strains

*ZRT2* upstream intergenic region deletions (*zrt2_US_* Δ/Δ), binding site mutants (*ZRT2_US_* AB^01^/AB^01^, *ZRT2_US_* AB^10^/AB^10^, and *ZRT2_US_* AB^11^/AB^11^), and wild-type add-back strains (*ZRT2_US_* AB^00^/AB^00^) were assayed for their abilities to grow on zinc-sufficient synthetic complete medium (2% dextrose, 0.17% yeast nitrogen base without ammonium sulfate, 0.5% ammonium sulfate, and auxotrophic supplements) and zinc-deficient medium (2% dextrose, 0.17% yeast nitrogen base without either ammonium sulfate or zinc sulfate, 0.2% ammonium sulfate, 2.5 μM EDTA, and auxotrophic supplements) on 2% agar plates (Nobile *etal.* 2009). Each strain was grown to saturation via overnight culture in YPD liquid medium at 30°C with shaking prior to back-dilution. Strains were grown to mid-log, washed, and then serial diluted in sterile phosphate-buffered saline (PBS). Two independent biological replicates for each engineered genotype were assayed twice. Aliquots of each dilution were spotted onto zinc-sufficient and zinc-deficient agar plates and grown at 30°C for 2 days.

### Biofilm phenotype assay

*C. albicans* strains were cultured from cryogenically frozen stocks at 30°C on YPD agar plates (2% bacteriological-grade peptone, 2% dextrose, 1% yeast extract, 2.5% agar) for 2 days. A single colony of each strain to be tested was grown overnight in liquid YPD medium. Biofilms were grown on the bottoms of 12-well polystyrene plates in Spider medium (1% nutrient broth, 0.2% K_2_HPO_4_, 1% mannitol, pH 7.2) with shaking at 200 rpm at 37°C using an ELMI shaker (ELMI) as described previously (Lohse *etal.* 2017; Gulati *etal.* 2018). The optical density 600 nm (OD_600_) was measured for each well using a Cytation 5 plate reader (BioTek), and biofilms were imaged. For each genotype, a *n* = 4 number of wells were assayed. Two independent biological replicates for each engineered genotype were assayed twice. Significance levels and confidence intervals were calculated by the Student t statistic with unequal variance using H_A_: OD_600_(+/+) ≠ OD_600_(AB/AB) and H_A_: OD_600_(+/+) > OD_600_(Δ/Δ).

### Software versions and computer specifications

The AddTag software is an open-source Python 3 software package developed for command line usage. We used AddTag r284 and AddTag r517 in Python 3.5.3 with the Regex 2018.2.21 package, and with the following additional software for scoring and aligning: Azimuth 2.0 (Doench *etal.* 2016) in Python 2.7.13 for on-target scores; CFD (Doench *etal.* 2016) and Hsu-Zhang (Hsu *etal.* 2013) for off-target scores; BLAST+ 2.7.1 (Camacho *etal.* 2009) for predicting recombination by aligning dDNA to gDNA; BOWTIE 2 2.3.4.1 (Langmead and Salzberg 2012) for aligning target sequences to off-target sites; MAFFT (Katoh *etal.* 2005) for identifying homologous flanking regions; and UNAFold 3.8 (Markham and Zuker 2008) for the change in Gibbs free energy and melting temperature thermodynamics calculations. We used the *C. albicans* assembly 22 sequence and annotations from the *Candida* Genome Database (http://www.candidagenome.org/) retrieved on February 05, 2017 (Skrzypek *etal.* 2017). Oligo designs were computed on a Linux 3.1.0 64-bit Slurm-managed 12-core (24-logical processors) system with 256 Gb RAM, with Intel Xeon E5-2650 v4 @ 2.20 GHz x86 CPU. Analyses were conducted using the bash shell (Free Software Foundation 2007). Full commands to reproduce the analysis are included in the code repository.

### AddTag software

Here, we provide an abbreviated overview of AddTag’s basic features. For additional details on target identification, feature expansion, scoring and ranking of targets, dDNA generation, and finding, scoring, and ranking primer designs, see Supplementary materials. A full list of available alternative target scoring algorithms, alignment programs, and thermodynamics calculators are detailed in the software documentation (https://github.com/tdseher/addtag-project).

The AddTag software requires users to input the full genome sequence (gDNA) of the organism to be edited, the start and end positions on a chromosome that will be edited (Feature), the sequence to which the feature will be modified (*Feature), and a description of the RGN to be used. AddTag also provides a variety of optional inputs that grant greater versatility, such as target motifs that define which sequences on the gDNA the RGN should interact with (Supplement—Identifying targets and feature expansion), and any extrinsic DNA sequence to replace the feature with when an edit is desired (Figures 1B and 4A). Finally, there are many other optional inputs including: the AddTag insert type (Supplement—Generating knock-out dDNAs that contain AddTag targets for intermediary genome) (Supplementary Figure S6); any specific Algorithms to rank target suitability (Supplementary Figures S7 and S8); a list of feature homologs; and parameters defining PCR conditions to optimize for (Supplementary Figure S9).

After processing the inputs, the AddTag software will produce four key results to both the terminal screen (STDOUT) and several text files: (a) A list of targets for restricting reference gDNA at the desired feature for initiating knock-out; (b) A list of Step one dDNA sequences that each contain a unique gRNA: RGN-binding site (AddTag target) with flanking segments homologous to regions upstream and downstream of the feature; (c) a Step two dDNA sequence that will restore the wild-type sequence or introduce a modified feature (*Feature); (d) A series of primers including AmpF/AmpR to amplify the Step two dDNA, shared sF/sR primers for assaying the feature across all steps of genome editing (+, Δ, and AB gDNAs), and iF/iR primers for positive amplification of the feature, and oF/oR primers to determine if the dDNA integration is at the correct locus. These validation (cPCR) primers all together indicate if the genomes were edited as intended.

### Data availability

Strains and plasmids are available upon request. All software and scripts used for this study are publicly-available at a Git repository hosted on GitHub (https://github.com/tdseher/addtag-project). This includes full documentation, source code, and example workflows. The authors affirm that all data necessary for confirming the conclusions of the article are present within the article, figures, and tables. Supplementary material is available at figshare: https://doi.org/10.25387/g3.14787933.

## Results

### The AddTag method enables precision genome editing of small features that do not overlap RGN target sites

To demonstrate how the two-step AddTag approach enables precision editing of small genomic features that cannot be targeted directly by traditional one-step methods without the introduction of undesired mutations, we modified three independent DNA binding sites for the Wor1 transcriptional regulator in *C. albicans* (*WOR2_DS_*, *WOR1_USd_*, and *WOR1_USp_*) (Figure 2A, Supplementary Figure S5, A and B) using our previously published *C. albicans* Cas9-mediated genome editing system (Nguyen *etal.* 2017). Although there are other *C. albicans* Cas9-based genome editing systems available (Vyas *etal.* 2015; Min *etal.* 2016; Huang and Mitchell 2017; Ng and Dean 2017), we chose to use the Nguyen *etal.* 2017 system since it can easily perform serial “markerless” genome edits. Briefly, this system relies on temporary integration of gRNA and Cas9 expression cassettes, along with a dominant selectable marker, within the *LEU2* gene. After confirming markerless integration of modified dDNA fragments at the target locus, the gRNA, Cas9, and resistance marker cassettes are removed via spontaneous recombination between flanking direct repeats and positive selection for restoration of the wild-type *LEU2* gene. This system yields precision homozygous genome edits with frequencies ranging from 30 to 100%, with the majority of transformations achieving at least 70% efficiency (Nguyen *etal.* 2017). We note that the fraction of colonies containing the intended modifications following the first and second steps of the AddTag process were comparable to those reported previously for the one-step approach, and since each step is an independent transformation, followed by isolation of strains with the intended genotype, the overall efficiency of editing is essentially identical between the one- and two-step AddTag methodologies. Wor1 is a well-characterized transcriptional regulator that binds to the consensus sequence TTAAAGTTT (Lohse *etal.* 2010; Hernday *etal.* 2013). Because this consensus sequence lacks an NGG PAM sequence, and the binding targets for Wor1 fall within A/T-rich intergenic regions, these Wor1-bound motifs would be challenging to edit using traditional one-step genome editing methods. In fact, the three Wor1 binding sites we selected not only lack an NGG PAM sequence, but they also lack any significant overlap with potential Cas9 target sites. Only one of these three Wor1 binding sites (*WOR1_USd_*) has any overlap with a potential Cas9 target site, however, the overlap lies within the final 4 bp of the gRNA target sequence (distal from the NGG PAM) and thus would likely require additional genome edits beyond the boundaries of the Wor1 binding site to enable direct (one-step) editing (Doench *etal.* 2016; Zheng *etal.* 2017). Looking more broadly, we find that the targeting constraints we observed with our three selected Wor1 binding sites are representative of all predicted Wor1-bound sites genome wide. Of the 352 predicted Wor1-bound sites, 217 (61.6%) have at least a single base pair of overlap with a potential Cas9 target site (20 bp gRNA target + NGG PAM), however, most of these features lack sufficient overlap with the potential Cas9 target sites to enable precision editing without introducing unwanted substitutions outside of the Wor1 binding sites. Upon filtering for sufficient overlap between the Cas9 target site and the Wor1-bound sites, as well as applying gRNA quality control thresholds to maximize on-target cutting and reduce off-target cutting, the number of Wor1-bound sites that could practically be edited by the direct one-step method is reduced to 0/352 (0%). This observation highlights the difficulty of performing targeted precision genome editing of this type of small A/T-rich genomic feature using traditional one-step methods.

For each of our three selected Wor1-bound motifs, we first deleted a region of the genome that includes each individual motif using a nearby high-quality RGN target site and replaced these regions with an AddTag target sequence that encodes a unique RGN target. In a subsequent round of transformations, the AddTag target sites were cut with Cas9 and the previously deleted regions were restored with either the wild-type genomic sequence (complementation) or a modified version in which the Wor1 consensus binding motif was replaced with a scrambled sequence (ACCCTTGCG). In all three cases, Sanger sequencing of PCR products spanning the edited loci revealed complete restoration of the wild-type sequence (complementation) or precise editing of the Wor1 binding motif (modification) without any unintended changes to the surrounding genomic DNA that was deleted and subsequently restored. Thus, we successfully demonstrated the ability of the AddTag methodology to precisely edit genomic loci that could not be edited via traditional (one-step) methods.

### The AddTag method can be used to streamline combinatorial editing of neighboring sites

To further demonstrate the utility of the AddTag strategy, we performed combinatorial editing of a pair of Zap1 transcription factor binding sites that are separated from each other by 645 bp within the upstream intergenic region of *ZRT2* in *C. albicans* (Figure 2B). Because these two binding sites are not immediately adjacent to each other, it would be extremely difficult to simultaneously edit both sites using traditional one-step genome editing methods (Supplementary Figure S10). Zap1 is a well-characterized zinc-finger transcriptional regulator that binds to the 11-mer DNA motif ACCTTNAAGGT (Zhao *etal.* 1998; Harbison *etal.* 2004; Nobile *etal.* 2009), and two instances of this motif are found centered under a peak of Zap1 binding upstream of the *ZRT2* gene, which encodes a major zinc transporter (Nobile *etal.* 2009). Because the two Zap1 binding sites upstream of *ZRT2* are separated by 645 bp, we deleted a minimal 668 bp region that encompassed both Zap1 binding sites and replaced this region with an AddTag target sequence (CGTACGCTGCAGGTCGACAGTGG). We next designed synthetic dDNA sequences to edit one, the other, or both of the Zap1 binding sites without altering any of the intervening sequence, and transformed these three independent mutant dDNAs, as well as a wild-type add-back version, into the *zrt2_US_* Δ/Δ base strain. The resulting series of *ZRT2_US_* add-back strains successfully restored the full wild-type sequence (*ZRT2_US_* AB^00^/AB^00^), mutated both the Zap1 binding sites (*ZRT2_US_* AB^11^/AB^11^), or individually mutated the CDS-proximal (*ZRT2_US_* AB^01^/AB^01^) or CDS-distal (*ZRT2_US_* AB^10^/AB^10^) Zap1 binding sites. Phenotypic assessment of these mutant strains revealed subtle yet consistent alterations in growth between each genotype that suggests that the promoter proximal site is required for Zap1-mediated activation of *ZRT2* on both zinc-sufficient and zinc-deficient media (Supplementary Figure S11).

### The AddTag method simplifies and improves gene complementation analyses

Gene deletion and complementation are two fundamental techniques in the reverse genetics approach to understanding gene function (Griffiths *etal.*  2000a, 2000b). However, typical complementation (add-back) approaches rely on gene expression from a nonnative locus, often in single copy, and thus are prone to issues with partial complementation or inconclusive results (Supplementary Figure S12) (Cheng *etal.* 2003; Staab and Sundstrom 2003; Brand *etal.* 2004; Burrack *etal.* 2016; Freire-Benéitez *etal.* 2016a, 2016b). To highlight the utility of our AddTag approach for gene complementation studies, and to demonstrate the power of creating homozygous gene add-backs at native loci, we performed gene deletions and add-backs for two key biofilm regulators in *C. albicans*. Biofilm formation is an important virulence trait of *C. albicans* that allows the fungus to successfully colonize host mucosal layers and cause local and disseminated disease in the host (Nobile and Johnson 2015). We chose to delete and subsequently restore two *C. albicans* genes (*EFG1* and *BRG1*) that encode master biofilm transcriptional regulators (Nobile *etal.* 2012). Under standard biofilm inducing conditions, strains with homozygous deletions (Δ/Δ) of either *EFG1* or *BRG1* have notably impaired biofilm growth (Nobile *etal.* 2012), while strains that are heterozygous (Δ/+) for either of these genes form biofilms that are intermediary between those produced by +/+ and Δ/Δ strains (Glazier *etal.* 2017). Previous studies showed that the traditional add-back approach, using a single-copy of either *EFG1* or *BRG1* integrated at a nonnative locus, failed to fully restore the wild-type biofilm phenotype, thus generating partial gene complementation results (Nobile *etal.* 2012).

Independent *EFG1* and *BRG1* homozygous gene deletion strains (*efg1* Δ/Δ or *brg1* Δ/Δ) were generated by replacing each CDS with unique minimal AddTag target sequences which were automatically designed by the AddTag software. Homozygous gene complementation strains were subsequently generated using gRNAs that direct Cas9 to cut the minimal AddTag target sequences, along with *EFG1 or BRG1* add-back dDNA sequences that were derived from PCR amplification of wild-type genomic DNA. To assess the phenotypes of the *EFG1* and *BRG1* deletion and add-back strains, relative to their wild-type counterparts, we performed a standard 24-hours biofilm growth assay (Lohse *etal.* 2017; Gulati *etal.* 2018) that assesses the extent of biofilm formation by optical density readouts (Figure 3, Supplementary Figure S13). The wild-type parental strain formed biofilms with the expected average OD_600_ of 0.69 ± 0.12 (mean ± standard deviation), while the *EFG1* and *BRG1* homozygous deletion strains yielded biofilms with OD_600_ values of 0.17 ± 0.02 and 0.29 ± 0.06, respectively, revealing severely compromised biofilm growth. Upon homozygous add-back of *EFG1* or *BRG1* into the respective deletion strains, robust biofilm growth that is statistically indistinguishable from that of the wild-type parental strain was observed (0.85 ± 0.06 for *EFG1_CDS_* AB/AB, and 0.83 ± 0.15 for *BRG1_CDS_* AB/AB). We note that the wild-type phenotype observed with these homozygous gene add-back strains stands in contrast to the previously reported add-back strains (single-copy add-back at a nonnative locus), which failed to fully complement the wild-type phenotype (Nobile *etal.* 2012). Together, these results demonstrate that the AddTag method can facilitate the generation of a complete set of matched isogenic strains to conclusively assess the phenotypic effects of specific gene deletions, without the ambiguity of partial complementation.

### AddTag software automates experimental designs for two-step genome editing

To facilitate implementation of this two-step genome editing methodology, we developed an AddTag software package that automates numerous critical experimental considerations that are necessary for successful two-step genome editing and genotypic verification. Users are required to make only broad decisions to provide the framework by which the program automates the experimental design process, thus decreasing the trial and error associated with gRNA target identification, dDNA design, and PCR primer selection. The software automatically identifies high quality RGN targets in the vicinity of the genomic feature to be edited (Supplementary Figure S14A), expands the selected feature to encompass an optimal RGN target site (Supplementary Figure S15), and designs dDNA fragments for both the first and second steps of editing (Supplementary Figure S14B). Furthermore, AddTag automatically generates an integrated minimal set of PCR primers that enable unambiguous genotypic verification at each step of the genome editing process (Figure 4, Supplementary Figure S16). All genome edits described in this study were successfully performed using AddTag-generated gRNA, dDNA, and PCR primer sequences, thus validating the utility of this automated design software. For a representative example of the PCR-based genotype verification assay, see Supplementary Figure S3.

## Discussion

Traditional one-step RGN-mediated genome editing procedures require modification of the RGN target site, and thus necessitate overlap between the RGN target and the feature that is being edited, or the introduction of undesired modifications outside of the feature of interest. By eliminating the need to modify the original RGN target site, the two-step genome editing methodology presented here is much more flexible, and enables a wide-range of genome editing applications that would otherwise be difficult to accomplish using traditional one-step approaches. A key capability of this methodology is the introduction of small-scale precision genome edits at loci that cannot be directly targeted by Cas9 (or your RGN of choice). Using the human fungal pathogen *C. albicans* as a test case, we demonstrate this capability by introducing targeted substitutions within three independent 9 bp transcription factor binding sites, all of which lack the necessary overlap with potential Cas9 target sites for traditional one-step editing. We also demonstrate that the AddTag approach enables facile combinatorial editing of loci that are proximal to each other in the genome, without the need to modify the intervening sequence. Furthermore, we restored previously deleted genes to their native loci, demonstrating how the AddTag approach facilitates improved gene complementation analyses without the need for molecular cloning. This native locus gene add-back approach resulted in complete restoration of the wild-type phenotype for the two gene deletion strains tested, whereas traditional methods had previously failed to achieve full phenotypic complementation for the same genes, further highlighting the advantages of the AddTag-mediated complementation method. While these examples are by no means exhaustive representations of the potential applications supported by the AddTag approach (other applications include, but are not limited to, the construction of translational fusions, construction of large mutant libraries, and modification of the lengths of repetitive elements), they highlight how this two-step process facilitates a wide-range of reverse genetic experiments by enabling seamless and efficient deletion and subsequent complementation or modification of virtually any locus in an organism that supports efficient RGN-mediated genome editing. We note that the overall efficiency of the two-step AddTag method is comparable to the traditional one-step method. Strains harboring the intended genome edits are isolated and verified by PCR analysis after each of the two steps, and thus the frequency of genome editing at the first step does not compound or reduce the efficiency of the subsequent second step.

Although we opted to edit nine-bp cis-regulatory motifs as a proof of concept for introducing small-scale targeted edits at sites that do not overlap RGN target sites, it should be possible to use the AddTag approach to change as little as a single base pair at virtually any site within the genome. We note that it could be possible to obtain some of these modifications via one-step editing using alternative RGNs, such as the SpG and SpRY variant of Cas9, which recognize “NGN” or “NRN” PAM sequences (Walton *etal.* 2020), or Cas12a, which recognizes a “TTTV” PAM sequence (Swarts and Jinek 2018). Many organisms (including *C. albicans*), however, lack well-characterized and efficient alternative RGN-mediated genome editing systems. A major advantage of the AddTag methodology is that it enables users to modify virtually any genomic locus using their preferred RGN, without the need for developing or optimizing alternative RGN-based genome editing systems. Another significant advantage, particularly when introducing small-scale edits, is that AddTag facilitates simple and unambiguous PCR-based screening for the desired genome modifications. Indeed, the removal and subsequent reintegration of at least a few hundred base-pairs of DNA during the first and second steps of the AddTag process generates substantial modifications to the genome that are easily assessed by standard PCR assays, whereas a small-scale edit produced via one-step editing can be challenging to discern from the wildtype via PCR analysis. While the AddTag methodology is flexible and facilitates otherwise challenging precision genome edits, there are some caveats and limitations that are worth considering when implementing this strategy. For example, this approach requires cutting at two distinct RGN target sites during two sequential steps of genome editing, whereas traditional approaches require only one round of cutting and HDR repair. While the extra round of cutting and repair increases the opportunity for unintended off-target cutting, we note that the AddTag approach and accompanying software enables the use of highly stringent gRNA selection criteria, which should significantly mitigate this risk. In contrast, when using the traditional one-step approach, particularly in the context of small-scale edits, one can often be faced with the decision of whether to proceed with a poor-quality gRNA that is more likely to result in off-target cutting, or to forego the desired experiment altogether. Although the AddTag approach uses the same pair of stringently selected RGN target sites to create homozygous wild-type and mutant “add-back” strains (step two) from the same base strain (step one), which further reduces the risk of confounding off-target effects, we cannot rule out the possibility that heterogeneity could arise as a result of off-target cutting; thus, we recommend assaying multiple independent isolates of any given strain. Another caveat of the AddTag approach is that heterozygosity within the region being edited (including any sequences that lie between the feature and target loci) can be lost. Indeed, we observed loss of heterozygosity within the 645 bp region between the two Zap1 binding sites during our combinatorial editing upstream of *ZRT2* (Supplementary Table S5). However, the potential effects of this loss of heterozygosity can be controlled for by performing Sanger sequencing of the affected region and selecting a matched set of wild-type add-back and mutant strains which are homozygous for the same allelic variant.

Overall, the integrated AddTag two-step genome editing strategy and supporting software package presented here provide a powerful, easily accessible, and customizable platform to facilitate unrestricted genome editing applications in organisms for which markerless RGN-mediated genome editing tools are available.
